# Editorial: Towards precision oncology: assessing the role of radiomics and artificial intelligence

**DOI:** 10.3389/fradi.2025.1676229

**Published:** 2025-09-03

**Authors:** Salvatore Claudio Fanni, Damiano Caruso, Lorenzo Faggioni, Emanuele Neri, Dania Cioni

**Affiliations:** ^1^ Department of Translational Research, Academic Radiology, University of Pisa, Pisa, Italy; ^2^ Radiology Unit, Department of Medical-Surgical Sciences and Translational Medicine, Sant'Andrea University Hospital, “Sapienza” University of Rome, Rome, Italy

**Keywords:** radiomics, machine learning, artificial intelligence, oncologic imaging, precision medicine

**Editorial on the Research Topic**
Towards precision oncology: assessing the role of radiomics and artificial intelligence

The paradigm of oncologic imaging is undergoing a profound transformation, driven by the increasing integration of radiomics and artificial intelligence (AI). Radiomics is a field within medical imaging that focuses on extracting a large number of quantitative features from medical images (1–3). Due to the high dimensionality of these features, radiomics particularly benefits from integration with AI techniques, especially machine learning, to identify meaningful patterns and support clinical decision-making (4, 5).

These technologies promise to enhance diagnostic precision, optimize therapeutic decision-making, and ultimately personalize patient management. The Research Topic “*Towards Precision Oncology: Assessing the Role of Radiomics and Artificial Intelligence*” brings together twelve contributions that collectively illuminate current advances and challenges in this rapidly evolving field.

This Research Topic spans a diverse range of clinical contexts, imaging modalities, and methodological approaches, reflecting the complexity and translational potential of AI in oncology.

Chest imaging is one of the most investigated topics, and it is not surprising that the majority of papers submitted and published in this research topic addressed the potential role of radiomics in this clinical scenario. Such interest of papers is very well represented in this research topic also thanks to a systematic review and meta-analysis published by Shi et al. The systematic review included 40 studies investigating radiomics in distinguishing between lung adenocarcinoma and lung squamous cell carcinoma. The area under summary receiver operating characteristic curve (SROC-AUC) for radiomics model based on CT, PET-CT and MR images were 0.86 (95% CI:0.82∼0.89), 0.85 (95% CI: 0.82∼0.88) and 0.79 (95% CI: 0.75∼0.82), respectively.

Zhang et al. developed a CT-based radiomics nomogram for solitary indeterminate smoothly marginated solid pulmonary nodules to differentiate benign from malignant ones. The authors implemented 19 radiomics features, one clinical variable (history of malignant tumor) and three semantic CT features (calcification, pleural retraction and lobulation) and achieved an outstanding area under the curve (AUC) of 0.93 in the validation cohort. Radiomics may also provide assistant in non-binary classification, such as in the diagnosis of the three histological subtypes of non-small cell lung cancer, i.e., adenocarcinoma, squamous cell carcinoma, and large cell carcinoma (LCC). Kuang et al. identified 9, 12 and 8 key features for the respective histological subtypes, and among the various machine learning models evaluated, XGB and Random Forest achieved the best performance.

Liu et al. adopted radiomics to differentiate lung adenocarcinoma from neuroendocrine neoplasm in a multicenter setting. The authors found that the merged model, combining both radiomics and semantic features, slightly outperformed both the radiomic model and semantic model. Once again, to combine different type of information may improve AI model performance.

However, lung cancer should be differentiated also from non-oncological entities, such as pulmonary granulomas. In this setting, to combine intra-nodular radiomic features with peri-nodular radiomic features may further improve AI models performance. Tian et al. extracted such radiomic features from lung adenocarcinomas and pulmonary granulomas on ^18^F-FDG PET/CT images and found that combining the two sets of features was better than using the intranodular model alone.

Another paper investigating the potential role of radiomics in differential diagnosis, in a different clinical scenario then lung cancer, is the one published by Hu et al. The authors investigated the potential role of a Rad-score to discriminate esophageal sarcomatoid carcinoma and esophageal squamous carcinoma, and found a 0.828 (95% CI 0.636–1.000) in the validation cohort.

Radiomics may also provide assistance in the characterization of tumoral lesions, which is of paramount importance to move radiology forward to precision oncology. Peng et al. evaluated the performance of a clinical-radiomics model to predict HER2 status in urothelial bladder carcinoma from contrast-enhanced CT images. Such clinical-radiomics model achieved an AUC of 0.85, and decision curve analysis indicated that the clinical-radiomics model provided good clinical benefit. Zhang et al. extracted radiomics from ultrasound images to predict tumor infiltrating lymphocyte levels in breast cancer. In comparison to grayscale ultrasound model, and radiomics model, the nomogram integrating both demonstrated superior discriminative ability on both the training (AUC 0.88) and testing (AUC 0.82) set. Ye et al. moved the field a step forward, as they compared a conventional radiomics model and a tumor internal heterogeneity habitat model in predicting pathologic complete response to neoadjuvant immunochemotherapy in non-small cell lung cancer. As a result, the habitat model exhibited higher AUC values compared to the conventional radiomics model, achieving an AUC of 0.78 and 0.72, respectively.

Moreover, although radiomic features are often described as imperceptible to the human eye, this does not imply that their conversion into colorimetric maps could not aid image interpretation and lesion detection. Hertel et al. used radiomics to improve detection of colorectal liver metastases on CT images and found that the feature map for firstorder RootMeanSquared was ranked superior in terms of very high visual contrast in 57.4% of cases, compared to 41.0% in standard reconstructions.

This very heterogeneous research topic also included papers investigating different clinical settings, such as the one by Kruzhilov et al., investigating the role of AI in whole-body PET imaging denoising, and the on by Tang et al., exploring the transformative application of the metaverse in nuclear medicine.

These studies demonstrate that the integration of radiomics and artificial intelligence represents a promising pathway to transform oncologic imaging into a quantitative and decision-support tool: from pulmonary nodules to molecular biomarkers, and toward new modalities for data visualization and optimization. However, to consolidate this transition into clinical practice, investment is needed in multicenter studies, standardized pipelines, model transparency, and interpretative interfaces. Such an approach could support the sustainable implementation of clinical AI and open new frontiers, including the educational and collaborative metaverse, in precision oncology.
